# The XpressCard Point-of-Care Test for Human Neutrophil Gelatinase-Associated Lipocalin Enhances the Prediction of Acute Kidney Injury

**DOI:** 10.3390/jcm13247564

**Published:** 2024-12-12

**Authors:** Kendra B. Bufkin, Jeane Silva

**Affiliations:** Department of Health Management, Economics and Policy, The Graduate School, Augusta University, Augusta, GA 30912, USA

**Keywords:** NGAL, acute kidney injury, AKI, biomarker, diabetes mellitus

## Abstract

**Background/Objectives:** Acute kidney injury (AKI), a common complication in hospitalized patients, is a clinical syndrome with a sudden and reversible decline in kidney function. Within hospitalization, the average incidence rate is 2% to 5%, and 67% of patients admitted to the Intensive Care Unit (ICU) acquire acute kidney injury. There is a pressing need to identify biomarkers that provide early detection to enhance the diagnosis of acute kidney injury. Neutrophil gelatinase-associated lipocalin (NGAL) has been identified as the most promising biomarker for predicting acute kidney injury due to upregulation as early as 2 h before kidney injury. **Methods:** Urine samples from 52 adult subjects were utilized to evaluate the accuracy of acute kidney injury diagnosis using the XpressCard for human NGAL. Sensitivity and specificity were calculated to analyze the validity of the NGAL XpressCard’s ability to accurately distinguish between acute and non-acute kidney injury. **Results:** The positive and negative predictive values were calculated to determine the prevalence of NGAL and predict the likelihood of diagnosing AKI. Data show that the NGAL XpressCard has a sensitivity and specificity of 59.3% and 95.8% and a positive and negative predictive value of 92.9% and 71.9%, respectively. **Conclusions:** The human NGAL XpressCard is effective at predicting AKI in hospitalized patients and correlates with high levels of HbA1c, which is associated with diabetes mellitus. It delivers immediate test results, which can enhance patient care.

## 1. Introduction

In recent years, the prevalence of AKI has significantly increased due to the continuing clinical and prognostic problems associated with this syndrome. It is estimated that more than 13 million people are affected by AKI annually, and 40% of those cases are accounted for by people diagnosed with diabetes mellitus (DM) [1]. DM is a major risk factor for AKI and leads to worse outcomes. In addition to AKI being more common in diabetic patients, they have a more severe outlook compared to those without DM [1] There are several proposed etiologies speculating why diabetes increases the risk of AKI, including chronic kidney disease, cardiovascular disease, heart failure, hyperglycemic crises, and medications used in the management of diabetes and other related comorbidities [2,3,4]. The diagnosis of AKI is challenging because of the lack of a sensitive and specific biomarker to measure kidney injury. Serum creatinine (SCr) and urine output are the standard diagnostic biomarkers to detect AKI; however, serum creatinine does not predict AKI quickly enough, leading to delayed diagnosis. Thus, there is a pressing need to identify a biomarker that can decrease the diagnostic window and detect structural injury instead of kidney damage. Identifying a better marker for AKI will help improve patient care and reduce healthcare costs. This article demonstrates that urinary NGAL (uNGAL) levels serve as indicators of kidney injury through neutrophil gelatinase-associated lipocalin (NGAL) XpressCard point-of-care testing at the early stages of AKI.

## 2. Methods

### 2.1. Study Design and Study Participants

This cross-sectional study was conducted at a Medical Center in East Central Georgia. The study enrolled adult patients aged 18 years or older who were admitted to the hospital and diagnosed with acute kidney injury according to KDIGO criteria (accessed on 17 November 2024, https://www.kidney-international.org/article/S0085-2538(23)00766-4/fulltext). The control kidney function group included healthy adults over 18 who were asymptomatic and visiting for routine medical check-ups. Exclusion criteria included individuals who already had chronic kidney disease and any of the following conditions: (1) serum creatinine above 6 mg/dL; (2) malignancy or infectious diseases; (3) severe proteinuria (>3.5 g/dL); (4) inflammatory states; (5) variations in leukocyte count; (6) renal transplant; and (7) undergoing treatment with steroids or immunosuppressant’s to minimize potential confounding factors. The Institutional Review Board reviewed and approved the study (IRB 1840264-2). An HIPAA waiver of authorization was granted to access subjects’ medical records and past and present health information.

### 2.2. Procedures

A diagnostic test was conducted on 52 adult patients to evaluate the presence of NGAL in controls and confirmed diagnosis of acute kidney injury subjects’ urine samples. Demographic and clinical characteristics were recorded in all subjects, including age, renal diagnosis, and medical history. Urine samples were collected into VACUETTE^®^ 6 mL urinalysis tubes or 8 mL urine analysis conical speckled tubes. The samples were stored at 2–8 °C until testing was performed with the human NGAL XpressCard (Antagen Pharmaceuticals, Boston, MA). All samples were analyzed within three days of their collection date, following the manufacturer’s instructions (https://www.antagen.net/wp-content/uploads/2015/05/NGAL-XpressCard-Antagenv1.pdf, accessed on 17 November 2024).

Briefly, cards were placed horizontally on the laboratory bench, 100 µL of urine was added to the sample wells, and the results were interpreted within 4 min. The concentration of uNGAL was assessed using the manufacturer’s sensitivity benchmarks to detect uNGAL levels. A weak band was interpreted as 50 ng/mL, a moderate band as 100 ng/mL, and a strong band as 1000 ng/mL, which was considered a positive test result. A negative outcome was indicated by the absence of an NGAL band on the testing card. These clinical diagnostic measurements were performed in triplicate.

### 2.3. Statistical Analysis

Using G-Power for Mac OS X (version 3.1.9.2; Universität Düsseldorf, Düsseldorf, Germany), a total sample size of 35 was needed to detect a large effect size and compare the difference between the two independent groups with 80% power at a 5% significance level and effect size of 0.8 [5]. An independent *t*-test was performed to determine if there was a significant difference between the means of the two groups. The Mann–Whitney U test was used to compare outcomes between groups for nonparametric variables. Pearson’s chi-squared and Fisher’s exact tests were used to determine whether there were any associations between categorical variables. Statistical significance was set at α = 0.05, and all data were analyzed using IBM SPSS Statistics for Macintosh Version 28.0 (IBM Corp, Armonk, NY, USA).

## 3. Results

A total of 52 participants were enrolled in this study at a medical center in East Central Georgia. Six subjects were excluded because of underlying medical conditions of leukocytosis (2), hyperkalemia (2), and heart disorders (2), leaving 46 subjects for analysis. Demographic information and clinical characteristics of the subjects are shown in Table 1.

We prospectively detected uNGAL protein expression levels in the remaining 46 patients using the human NGAL XpressCard. The independent sample *t*-test was conducted to compare statistical significance between AKI and control variables, such as age, height, BMI, laboratory values of blood urea nitrogen (BUN), SCr, estimated glomerular filtration rate (eGFR), and glycated hemoglobin (HbA1c), and length of hospital stay (LoS). There were significantly higher values for the AKI group compared to the control group for age (*t* (44) = −2.89, *p* = 0.006), BUN (*t* (34) = −4.34, *p* < 0.001), SCr (*t* (32) = −4.06, *p* < 0.001), and LoS (*t* (44) = −3.11, *p* = 0.003). On the other hand, eGFR was significantly lower in the AKI group compared to the control group (*t* (33) = 5.73, *p* < 0.001). The means of the two groups were significantly different for age, BUN, SCr, LoS, and eGFR (Table 1). Fisher’s exact test was used to determine if there was a significant association between hospitalization and the diagnosis established. The two variables had a statistically significant association (two-tailed *p* ≤ 0.001). All data were analyzed using IBM SPSS Statistics for Macintosh Version 28.0 (IBM Corp, Armonk, NY, USA).

**Figure 1:**
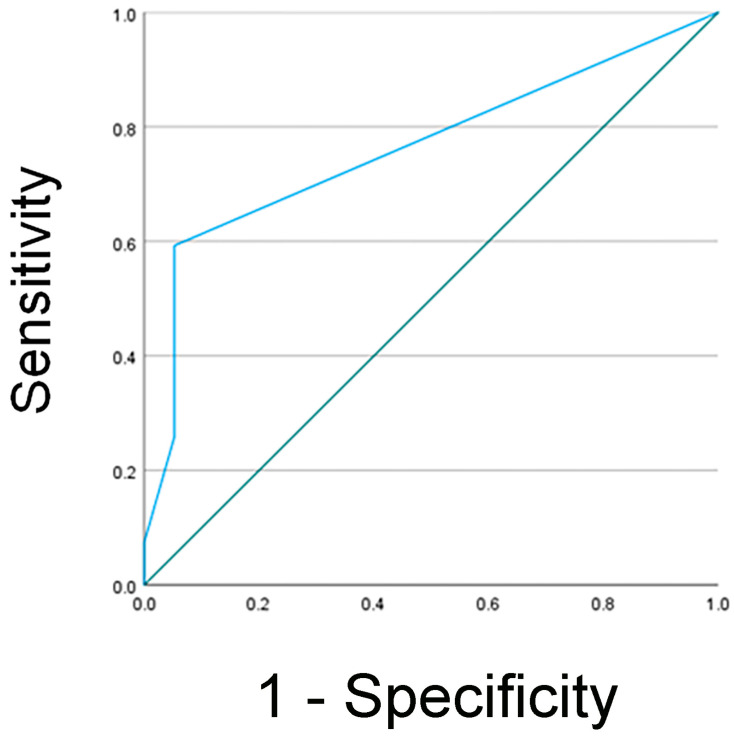
Analysis of receiver operating characteristic curve for uNGAL as a predictive biomarker in AKI. The area under the curve was estimated as 0.763 ± 0.072, *p* = 0.003. At the 25.00 cutoff value, the sensitivity was 59.3%, and the specificity was 95.8%.

NGAL Detection and Clinical Outcome: To determine the association between clinical outcomes and NGAL detection via the human NGAL XpressCard in confirmed AKI diagnosis using KDIGO criteria, we determine the sensitivity, specificity, negative predictive value, and positive predictive value for AKI. Receiver operating characteristic (ROC) curve analysis was performed to identify the optimal cutoff value of uNGAL levels, sensitivity, specificity, and positive and negative predictive value for the prediction of AKI (Figure 1). The area under the curve (AUC) was calculated to assess the test’s accuracy, resulting in a value of 0.763 ± 0.072, with *p* = 0.003. Using a cutoff value of 25.00, the sensitivity and specificity of uNGAL for detecting acute injury were 59.3% and 95.8%, and positive and negative predictive values were 92.9% and 71.9%, respectively. Of the 22 patients with confirmed AKI diagnosis, uNGAL protein expression levels were present in 59.3% of the subjects (13/22).

**Figure 2:**
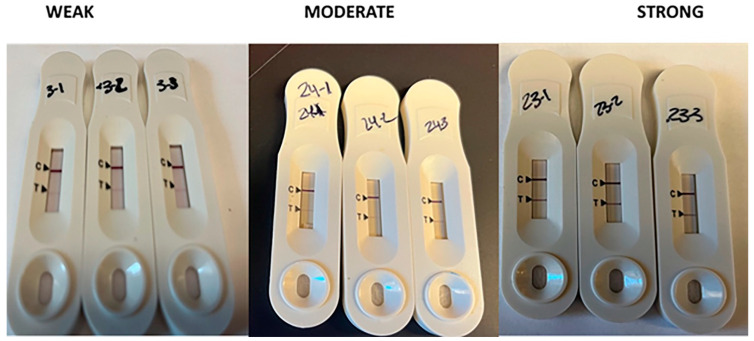
NGAL band intensity classification. The estimated concentration of uNGAL was based on the assay’s sensitivity to detect uNGAL, which is as follows: weak = 50 ng/mL, moderate = 100 ng/mL, and strong = 1000 ng/mL. The assay is sensitive to detect NGAL down to 50 ng/mL. Any density below this threshold was considered negative.

The intensity of the uNGAL band is reported as weak, moderate, or strong (Figure 2). Eight patients demonstrated weak intensity, four had moderate band intensity, and only one showed strong NGAL expression. Of the 13 patients with uNGAL levels detected, 11% were admitted to the hospital, 9% had diabetes mellitus, 8% had hypertension, and 5% were diagnosed with coronary artery disease. An independent sample *t*-test was performed to compare age, height, BMI, urine and serum biomarkers BUN, SCr, eGFR, and HbA1c levels, and length of hospital stay with the clinical outcome of having uNGAL detected, and these tests did not reach statistical significance. The results indicated that patients diagnosed with AKI are often critically ill, leading to a longer stay in the intensive care unit. A subset of this population is also diagnosed with cardiovascular diseases, including hypertension and coronary artery diseases. Additionally, these patients have an average HbA1c level of 9.2 ± 3.8, which is associated with type 2 diabetes.

These findings show that NGAL is detected in the urine of patients with AKI using human NGAL XpressCard point of care, ranging from weak to strong intensity levels, and can be used as a potential biomarker to predict AKI.

## 4. Discussion

The standard diagnostic tools for AKI detection are SCr and urine output, markers of renal function. Because these markers do not indicate the severity of the damage until it is too late to reverse the process, a more effective biomarker to predict kidney injury is urgently needed. In this study, NGAL was investigated as a potential prognostic biomarker for AKI to overcome the limitations of using SCr and urine output as a diagnostic tool for AKI.

Common comorbidities associated with acute kidney injury are cardiovascular disease, heart failure, CKD, and diabetes [6]. In this study, the data showed that AKI was more common in hospitalized patients, those with extended length of stay, and patients with high HbA1c levels, which is associated with type 2 diabetes. There have been many reasons postulated as to why diabetes increases the risk of AKI, including hyperglycemic crises, medications, chronic kidney diseases, and heart failure. Hyperglycemic complications of diabetes, such as diabetic ketoacidosis or hyperosmolar hyperglycemic states, increase the risk of AKI because it is associated with dehydration or rhabdomyolysis complications, which increase the progression of AKI [2]. Data have shown that AKI develops in 26% of patients undergoing coronary artery bypass grafting with heart failure and is more common among those with diabetes [7] and patients with hypertension and coronary artery disease. The renin–angiotensin–aldosterone system (RAAS) is frequently used in the treatment of hypertension and to help slow kidney disease progression. However, the RAAS promotes AKI development by reducing intraglomerular pressure via vasodilation of the efferent arteriole [2]. All these comorbidities associated with diabetes lead to the development of AKI and consequently kidney failure. The severity of this medical condition highlights the need for a reliable predictive biomarker that can specifically diagnose AKI at an earlier stage. Such biomarkers would enhance decision-making in the treatment of AKI. However, a validated biomarker for predicting AKI has yet to be identified.

This study provides evidence that NGAL is detected early in subjects with AKI and is effective in predicting disease progression. However, AKI can be predicted more accurately by combining uNGAL with markers such as urinary output and serum creatinine (SCr), which are still the gold standard for diagnosing AKI.

The present study has limitations that should be reported. First, it was a single-center study with a relatively small cohort, and NGAL detection was verified by a qualitative method. These limitations did not allow for a more comprehensive evaluation of AKI progression or ROC analysis to determine whether NGAL predicts AKI better than other biomarkers, such as SCr. Confirmation in larger cohorts will attribute general validity to our study. However, further in-depth examinations should be performed to validate urinary NGAL as a biomarker to predict and monitor AKI using the human NGAL XpressCard. In conclusion, the data showed that the human NGAL XpressCard is reliable and provides rapid test results that enhance patient care.

## Figures and Tables

**Table 1:** Patient demographic and clinical characteristics based on the established diagnosis.

Variable	All Patients (*n* = 46)	Normal Kidney Function (*n* = 24)	AKI (*n* = 22)	*p* Value
Age, years	42.7 ± 17.5	36.2 ± 14.6	50 ±17.8	0.006 *
Sex				
Female	26.0 (56.5%)	16.0 (66.7%)	10.0 (45.5%)	0.147
Male	20.0 (43.5%)	8.0 (33.3%)	12.0 (54.5%)	
Race				
Black	18.0 (39.1%)	8.0 (33.3%)	10.0 (45.5%)	0.300
White	25.0 (54.3%0	13.0 (54.2%)	12.0 (54.5%)	
Other	3.0 (6.5%)	3.0 (12.5%)	0.0 (0.0%)	
Height, cm	169.2 ± 10.4	168.1 ± 10.4	170.3 ± 10.5	0.478
Weight, lb	169.4 (146.5–208.2)	173.4 (137.2–210.7)	165.0 (149.9–199.7)	0.991
BMI, kg/m^2^	28.5 ± 8.1	28.7 ± 7.6	28.3 ± 8.8	0.842
Laboratory Data				
BUN, mg/mL	22.2 ± 17.6	9.1 ± 5.4	30.5 ± 17.7	<0.001 *
SCr, mg/dL	1.7 ± 1.2	0.8 ± 0.2	2.2 ± 1.2	<0.001 *
eGFR, mL/min/1.73 m^2^	58.8 ± 29.9	85.9 ± 7.8	42.8 ± 26.3	<0.001 *
HbA1c, %	9.2 ± 3.8	------	9.2 ± 3.8	------
Hospitalization	23.0 (50%)	4.0 (16.7%)	19.0 (86.4%)	<0.001 *
Length of hospital stay, day	3.2 ± 6.1	0.8 ± 0.7	5.9 ± 8.0	0.003 *

Data are expressed as means ± SD, median (25–75% interquartile range) for nonparametric variables, or number (percentage). * *p* < 0.05.

## Data Availability

All data generated or analyzed during this study are included in this published article.

## List of Abbreviations

AKI	acute kidney injury
AUC	area under the curve
BUN	blood urea nitrogen
DM	diabetes mellitus
eGFR	glomerular filtration rate
HbA1c	glycated hemoglobin
LoS	length of hospital stay
ICU	intensive care unit
NGAL	neutrophil gelatinase-associated lipocalin
RAAS	renin–angiotensin–aldosterone system
ROC	receiver operator characteristics
SCr	serum creatinine
uNGAL	urinary NGAL
